# Ocular Hypotensive Effects of the ATP-Sensitive Potassium Channel Opener Cromakalim in Human and Murine Experimental Model Systems

**DOI:** 10.1371/journal.pone.0141783

**Published:** 2015-11-04

**Authors:** Uttio Roy Chowdhury, Cindy K. Bahler, Bradley H. Holman, Peter I. Dosa, Michael P. Fautsch

**Affiliations:** 1 Department of Ophthalmology, Mayo Clinic, Rochester, MN, United States of America; 2 Institute for Therapeutics Discovery and Development, University of Minnesota, Minneapolis, MN, United States of America; Casey Eye Institute, UNITED STATES

## Abstract

Elevated intraocular pressure (IOP) is the most prevalent and only treatable risk factor for glaucoma, a leading cause of irreversible blindness worldwide. Unfortunately, all current therapeutics used to treat elevated IOP and glaucoma have significant and sometimes irreversible side effects necessitating the development of novel compounds. We evaluated the IOP lowering ability of the broad spectrum K_ATP_ channel opener cromakalim. Cultured human anterior segments when treated with 2 μM cromakalim showed a decrease in pressure (19.33 ± 2.78 mmHg at 0 hours to 13.22 ± 2.64 mmHg at 24 hours; p<0.001) when compared to vehicle treated controls (15.89 ± 5.33 mmHg at 0 h to 15.56 ± 4.88 mmHg at 24 hours; p = 0.89). In wild-type C57BL/6 mice, cromakalim reduced IOP by 18.75 ± 2.22% compared to vehicle treated contralateral eyes (17.01 ± 0.32 mmHg at 0 hours to 13.82 ± 0.37 mmHg at 24 hours; n = 10, p = 0.002). Cromakalim demonstrated an additive effect when used in conjunction with latanoprost free acid, a common ocular hypotensive drug prescribed to patients with elevated IOP. To examine K_ATP_ channel subunit specificity, K_ir_6.2^(-/-)^ mice were treated with cromakalim, but unlike wild-type animals, no change in IOP was noted. Histologic analysis of treated and control eyes in cultured human anterior segments and in mice showed similar cell numbers and extracellular matrix integrity within the trabecular meshwork, with no disruptions in the inner and outer walls of Schlemm’s canal. Together, these studies suggest that cromakalim is a potent ocular hypotensive agent that lowers IOP via activation of K_ir_6.2 containing K_ATP_ channels, its effect is additive when used in combination with the commonly used glaucoma drug latanoprost, and is not toxic to cells and tissues of the aqueous humor outflow pathway, making it a candidate for future therapeutic development.

## Introduction

Glaucoma is a complex, multifactorial ocular neuropathy and the leading cause of irreversible blindness, causing vision loss to millions of people worldwide.[1, 2] As the world population continues to age, the prevalence of glaucoma will increase and is estimated to reach over 110 million by 2040.[3] Primary open angle glaucoma is the most common form of the disease with over 80% prevalence rate among glaucoma patients in the United States.[4]

Despite recent advances in glaucoma research, the biology and pathophysiology behind the development of glaucoma remains poorly understood.[2, 5] Since glaucoma lacks good reference standards for diagnosis and can be asymptomatic until advanced stages, early detection of the disease can be challenging.[2, 5] Elevated intraocular pressure (IOP) is the most prevalent and only treatable risk factor for the disease.[5, 6] Various classes of drugs that are commonly used to reduce IOP include prostaglandin analogues (e.g. latanoprost), α2 adrenergic agonists (e.g. brimonidine), β blockers (e.g. timolol), carbonic anhydrase inhibitors (e.g. dorzolamide) and cholinergic agonists (e.g. pilocarpine).[5, 7] Unfortunately, all these drugs have significant and sometimes irreversible side effects.[5, 8] For example, prostaglandin analogues cause hypertrichosis and permanent darkening of the iris and eyelashes; [9, 10] α2 adrenergic agonists can cause allergic conjunctivitis and hyperemia;[11] β blockers have detrimental cardiovascular as well as corneal effects;[12, 13] and cholinergic agonists can cause headaches due to ciliary spasm, blurring of vision and induce myopia.[2, 5, 14] As a result, continued research aimed at developing novel pharmacologic agents for the treatment of glaucoma is a priority.[15]

Our laboratory has identified a novel class of ocular hypotensive agents in the form of ATP-sensitive potassium (K_ATP_) channel openers. K_ATP_ channels are a group of unique hetero-octameric proteins that are affected by changes in micromolar concentrations of intracellular ATP. As a result, these channels connect the metabolic and energetic state of cells.[16–18] Although first identified in cardiac muscle cells,[19] K_ATP_ channels have subsequently been described in skeletal[20] and smooth muscle,[21] pancreatic beta cells,[22] as well as neurons.[23] The K_ATP_ channels are involved in the regulation of several vital cellular functions including protection against cellular swelling,[24–26] various stress factors (shear, stretch, oxidative etc.) and shock.[17, 18, 27–38] These channels are also involved in the formation of gap and tight junctions.[39–41]

K_ATP_ channel openers are already in clinical use for various non-ocular disorders including systemic hypertension and ischemic heart diseases.[42–45] Recently, we have shown that several major K_ATP_ channel openers (e.g. diazoxide, nicorandil) have ocular hypotensive properties in human and animal model systems.[46, 47] Cromakalim is a benzopyran compound that is regarded as a prototypical K_ATP_ channel opener.[48, 49] Based on our previous studies with K_ATP_ channel openers, we would expect cromakalim to have similar ocular hypotensive effects to diazoxide and nicorandil. However, cromakalim has been reported to increase IOP.[50] In light of this, we evaluated the effect of cromakalim on IOP in human anterior segment organ culture and murine experimental model systems.

## Methods

### Human anterior segment perfusion culture

Use of human donor eyes was approved by the Mayo Clinic Institutional Review Board (IRB 06–005928) and conformed to the ethical guidelines laid down by the Declaration of Helsinki. A total of 17 donor eyes [12 males and 5 females; age 70.78 ± 14.96 years, mean ± standard deviation (SD); age range, 40–93 years] were obtained from the Minnesota Lions Eye Bank post mortem and cultured as previously described. [46, 51] Briefly, the eyes were bisected at the equator and the lens, ciliary body, iris, vitreous humor and the posterior half of the eye with the retina were discarded. The anterior segments were clamped in modified petri dishes and perfused with Dulbecco’s modified Eagle’s media (DMEM; Mediatech, Manassas, VA) containing 1% antibiotic/antimycotic solution (Sigma, St. Louis, MO). Anterior segments were perfused at the normal human aqueous humor flow rate (2.5 μl/min) and maintained at 37°C in 5% CO_2_. Using an automated and custom designed software system, pressure inside the anterior chamber was monitored in real time through a secondary access cannula built into the modified petri dishes.

#### Treatment with cromakalim

For each pair of cultured eyes, one eye was perfused with 0.02 (n = 5), 0.2 (n = 3) or 2.0 μM (n = 9) cromakalim (diluted in DMEM from a stock concentration of 2 mM in DMSO) while the contralateral eye received vehicle (DMSO, diluted in DMEM). Both vehicle and drug were added by anterior chamber exchange using a gravity driven, constant pressure method over a 5 minute period. Following anterior chamber exchange, pressure was recorded hourly for a period of 24 hours. Each hourly recording was an average of 60 one-minute pressure measurements. Following termination of perfusion with cromakalim, outflow facility (C) was calculated at 0 hour (C_0_) and 24 hours (C_d_) after addition of cromakalim by dividing the flow rate (2.5 μl/ min) by the corresponding pressure reading at a given time point. Percent change in outflow facility was obtained by the formula [(C_d_/C_0_) - 1] X 100.[46] Percent changes in outflow facility for both treated and control anterior segments of all pairs were averaged and expressed as mean ± standard deviation.

### Animals

All animal studies adhered to the tenets of the ARVO Statement for the Use of Animals in Ophthalmic and Vision Research. All protocols utilizing animals were reviewed and approved by the Mayo Clinic Institutional Animal Care and Use Committee (IACUC A3414, A67713, A42713). Wild type C57BL/6 mice (retired breeders, age >8 months, weight around 30 gm) were purchased from Charles River Laboratories (Wilmington, MA). K_ir_6.2^(-/-)^ mice were a generous gift from Dr. Andre Terzic (Mayo Clinic, Rochester, MN). All mice were maintained in the Mayo Clinic Animal Care Facility under a 12 hour light and 12 hour dark cycle and received water and food *ad libitum*. All animals were acclimatized to housing conditions for at least 7 days before experiments were initiated. Following completion of experiments, mice were sacrificed by CO_2_ exposure mediated through a rodent euthanasia machine (REM), as per IACUC guidelines. Briefly, animals were placed in designated CO_2_ chambers (maintained for this purpose by the Department of Comparative Medicine, Mayo Clinic, Rochester, MN) and the REM was started to initiate a 4 stage automatic control of CO_2_ inflow. The REM proportionately increases the CO_2_ concentration across the stages while allowing intermittent “wait times” for the gas to take effect. Ocular enucleations were performed within 10 minutes of death.

### Animal treatment and measurement of IOP

IOP was measured in live, conscious mice using a hand held rebound tonometer (Icare Tonolab; Colonial Medical Supply, Franconia, NH). Prior to initiation of experiments, mice had IOPs measured daily for at least 3 days, to habituate the animals to handling and pressure recording procedures. Briefly, IOP was measured by striking the center of the cornea with the tonometer probe.[47] Following six readings, average IOP was calculated by the tonometer by discarding the highest and the lowest values. This average was considered one IOP reading. Average of three independent readings was recorded as the IOP at a given time point.

#### Pretreatment

IOP was measured at 3 different time points every day for 3 consecutive days. These timepoints corresponded to designated treatment timepoints as described below. The average IOP of the three time points was recorded as the daily IOP.

#### Cromakalim Treatment

Cromakalim (Sigma-Aldrich; St. Louis, MO) was prepared as a stock solution of 100 mM in DMSO since cromakalim has limited solubility in aqueous solution. A working concentration of 5 mM cromakalim was prepared by diluting the stock 20 fold in 10% Cremophor EL (Sigma-Aldrich) in PBS. Addition of Cremophor EL helped maintain the cromakalim in solution and because it is a surfactant, helped permeabilize the cornea for better drug penetration following topical application. For treating mice (wild type, n = 10; K_ir_6.2^(-/-)^, n = 10), a 5 μl bolus of 5 mM cromakalim was added topically to one eye once daily for 5 consecutive days. The contralateral eye was treated with an equivalent amount of vehicle in 10% Cremophor EL without cromakalim. IOP was measured in treated and control eyes at 1, 4 and 23 hours following treatment. Average IOP from these three time points was recorded as the daily IOP.

#### Combination treatment with cromakalim and latanoprost free acid (LFA)

LFA (purity ≥ 98%; Cayman Chemicals, Ann Arbor, MI) was supplied as a solution in methyl acetate. Methyl acetate was evaporated in a gentle stream of nitrogen. LFA was reconstituted in DMSO and purged with nitrogen for a stock concentration of 0.1 M. This stock solution was diluted 1000 fold in the working solution of cromakalim (described above) to obtain a final LFA concentration of 0.1 mM, similar to the concentration of latanoprost in the commercially available drug Xalatan that is used to treat ocular hypertension in humans. For mouse studies, 5 μl of cromakalim/LFA cocktail (containing 5 mM of cromakalim and 0.1 mM LFA) was added to one eye of the animal while the contralateral eye received DMSO and Cremophor EL in equivalent proportions to the treated eye. Daily combination treatment with both cromakalim and LFA was continued for 5 days. IOP was measured at 1, 4 and 23 hour time points following treatment as described above.

#### Post treatment

IOP recording was continued at similar time points following cessation of treatment. Post treatment IOP recording was continued for 3 consecutive days.

### Histology

Following completion of the human anterior segment perfusion culture experiments, two tissue wedges 180° apart that included the trabecular meshwork (TM) and Schlemm’s canal (SC) from all anterior segments (n = 17 pairs), were isolated and fixed in 4% paraformaldehyde in 0.1 M phosphate buffer (pH 7.2). The wedges were post fixed in 2% osmium tetraoxide (Electron Microscopy Sciences, Hatfield, PA) in 0.1 M phosphate buffer and dehydrated in an ascending series of ethanol. The tissues were transferred to a clearing agent (propylene oxide, Electron Microscopy Sciences), embedded in epoxy resin blocks and 0.5 μm and 0.1 μm sections were cut using an ultramicrotome (Leica Microsystems, Buffalo Grove, IL). The 0.5 μm sections were stained with toluidine blue for assessment of general morphology by light microscopy. The 0.1 μm sections were put on copper grids and stained with 2% uranyl acetate (Electron Microscopy Sciences) followed by lead citrate (Mager Scientific, Dexter, MI). Representative 0.1 μm sections from 6 eye pairs were analyzed using a transmission electron microscope (JEOL 1400; JEOL USA, Peabody, MA).

For mouse eyes, following end of treatment, eyes were enucleated and fixed in 4% paraformaldehyde in 0.1 M phosphate buffer. Similar procedures for transmission electron microscopy were followed as described above for human eyes.

### Statistics

All values are expressed as mean ± standard deviation. Distribution of data sets was evaluated using Shapiro-Wilk test. IOP between experimental and vehicle treated control eyes was compared using Student’s paired t-test for data sets with normal distribution and Wilcoxon sign-rank test for non-parametric data sets. Bonferroni correction was applied to compensate for multiple comparisons. Differences were considered significant when p ≤ 0.05. For analysis corrected for multiple comparisons, significance for p value was changed to ≤ 0.045 (cromakalim treatment) and to ≤0.003 (cromakalim + LFA combination treatment). Variations in daily IOP is graphically presented as difference between the treated and vehicle control eyes (ΔIOP). Statistical calculations were performed using JMP statistical software package (SAS, Cary, NC)

## Results

### Cromakalim lowers pressure and increases outflow facility in *ex vivo* perfusion cultures of human anterior segments

**Fig 1:**
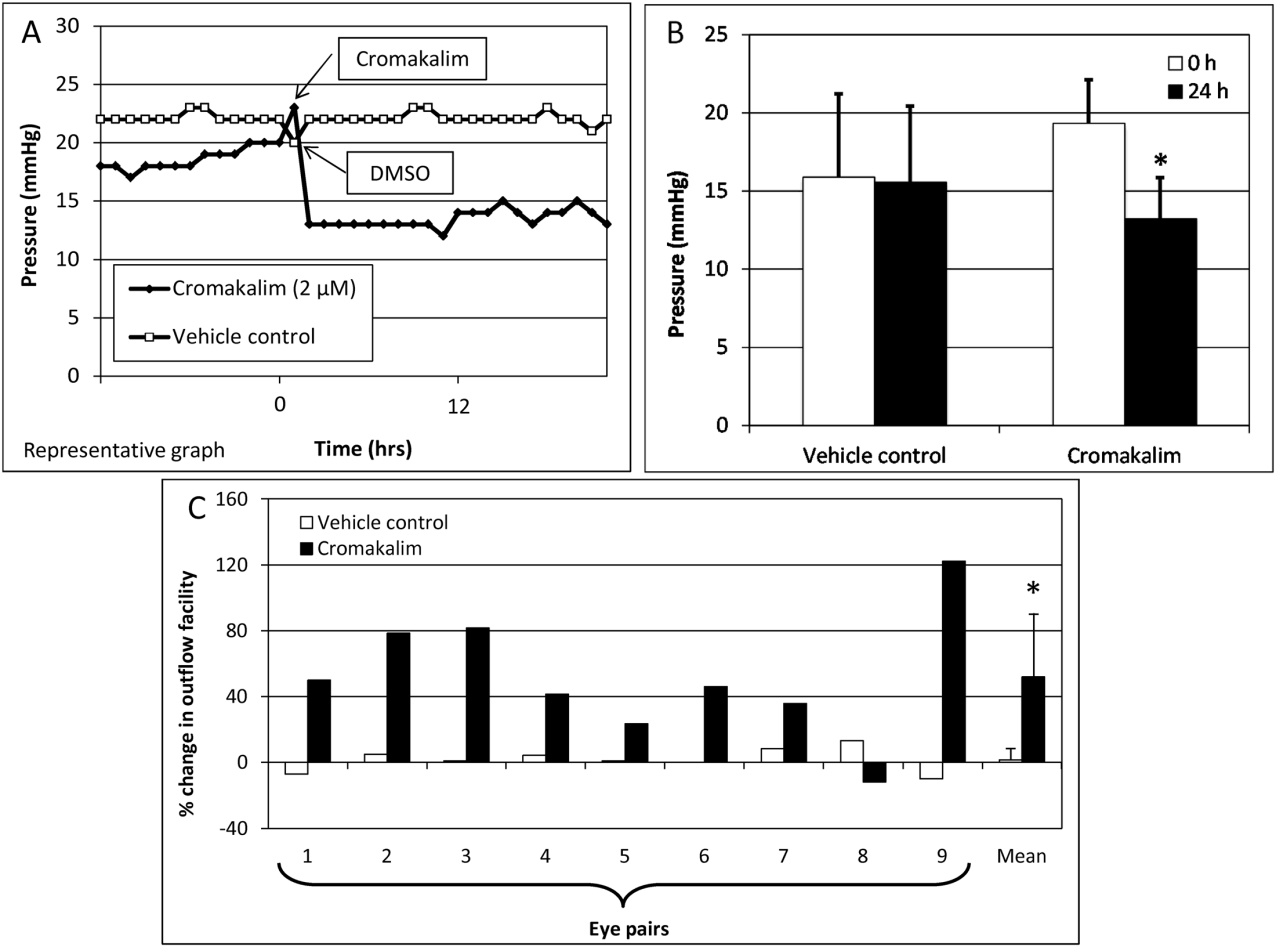
The K_ATP_ channel opener cromakalim has ocular hypotensive activity in perfusion cultures of human anterior segments. A) Representative graph shows reduction in pressure following addition of cromakalim (2 μM). B) Treatment with 2 μM cromakalim caused significant decrease in pressure when compared to vehicle (DMSO) treated controls (n = 9). C) Outflow facility in all eye pairs treated with cromakalim for 24 hours along with the mean value for the group. Outflow facility was increased in 8 out of the 9 eye pairs. Data represents mean ± standard deviation. *p<0.001.

To evaluate the hypotensive effects of the broad spectrum K_ATP_ channel opener cromakalim, anterior segments of human donor eyes were perfusion cultured and following attainment of stable baseline pressure, different concentrations of cromakalim were added to one eye while the contralateral eye received vehicle in the same proportion. Treatment with 0.02 (n = 5) and 0.2 μM (n = 3) for 24 hours showed no consistent pressure change compared to baseline (0.02 μM: 16.40 ± 2.07 mmHg at 0 hour vs. 16.20 ± 5.02 mmHg at 24 hours, n = 5, p = 0.94; 0.2μM: 18.67 ± 4.62 mmHg at 0 hour vs. 19.00 ± 4.36 mmHg at 24 hours, n = 3, p = 0.42). With 2 μM cromakalim, pressure inside the anterior chamber started to drop within one hour of drug treatment and reached maximal reduction within 3–4 hours (Fig 1A). The pressure was maintained at this reduced level for the entire duration of the experiment. Treatment with 2 μM cromakalim caused a reduction in pressure from 19.33 ± 2.78 mmHg at 0 hour to 13.22 ± 2.64 mmHg at 24 hours (reduction of 31.61%; p<0.001; n = 9), while pressure in the vehicle treated eyes remained unchanged during the same time period (15.89 ± 5.33 mmHg at 0 hour to 15.56 ± 4.88 mmHg at 24 hours, p = 0.89.0; n = 9) (Fig 1B). Outflow facility increased in 8 out of the 9 eyes treated with 2 μM cromakalim (Fig 1C). On average, outflow facility increased from 0.13 ± 0.02 μl/min/mmHg at 0 hour to 0.20 ± 0.04 μl/min/mmHg at 24 hours (48.99% increase, p<0.05). In contrast, vehicle treated anterior segments showed no change in outflow facility which was maintained at 0.17 ± 0.06 μl/min/mmHg at 0 hour and 0.17 ± 0.05 μl/min/mmHg at 24 hour time points (n = 9, p = 0.92) (Fig 1C).

**Fig 2:**
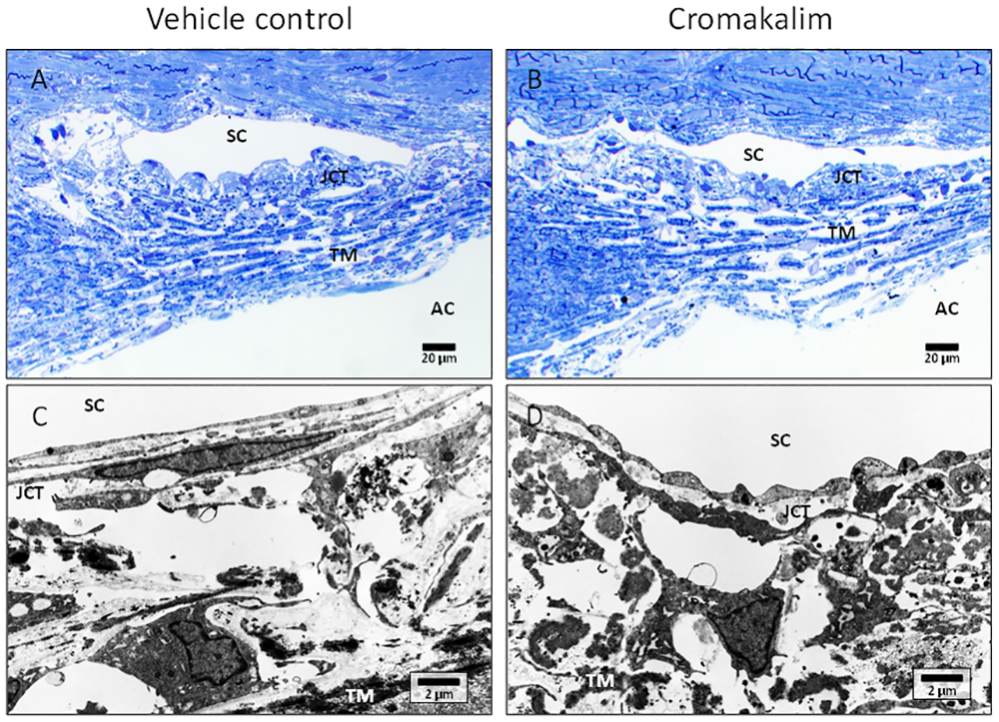
Histologic analysis of human anterior segment ocular tissue following treatment with cromakalim. A, B) 1 μm toluidine blue stained representative sections of vehicle (DMSO) and cromakalim treated eyes. C, D) Transmission electron micrographs showing ultrastructure of vehicle and cromakalim treated eyes. Vehicle and treated eyes had similar morphology and ultrastructural appearance showing no detrimental side effects of cromakalim treatment. SC = Schlemm’s canal; TM = trabecular meshwork; AC = anterior chamber; JCT = juxtacanalicular region.

Following termination of experiment, all eyes were examined histologically to evaluate potential side effects of the drug on the conventional outflow pathway and to confirm presence of healthy TM. Both vehicle and cromakalim treated eyes showed normal juxtacanalicular (JCT) region and trabecular meshwork, with viable cells populating the trabecular beams, as evident from toluidine blue stained 0.5 μm sections (Fig 2A and 2B) and transmission electron micrographs (Fig 2C and 2D). The Schlemm’s canal in control and treated eyes was open with intact inner and outer walls. Overall, there were no observable changes in the appearance of the outflow pathway, indicating that cromakalim treatment did not cause any significant changes to cell or tissue morphology.

### Ocular topical administration of cromakalim lowered IOP in an *in vivo* normotensive murine model

**Fig 3:**
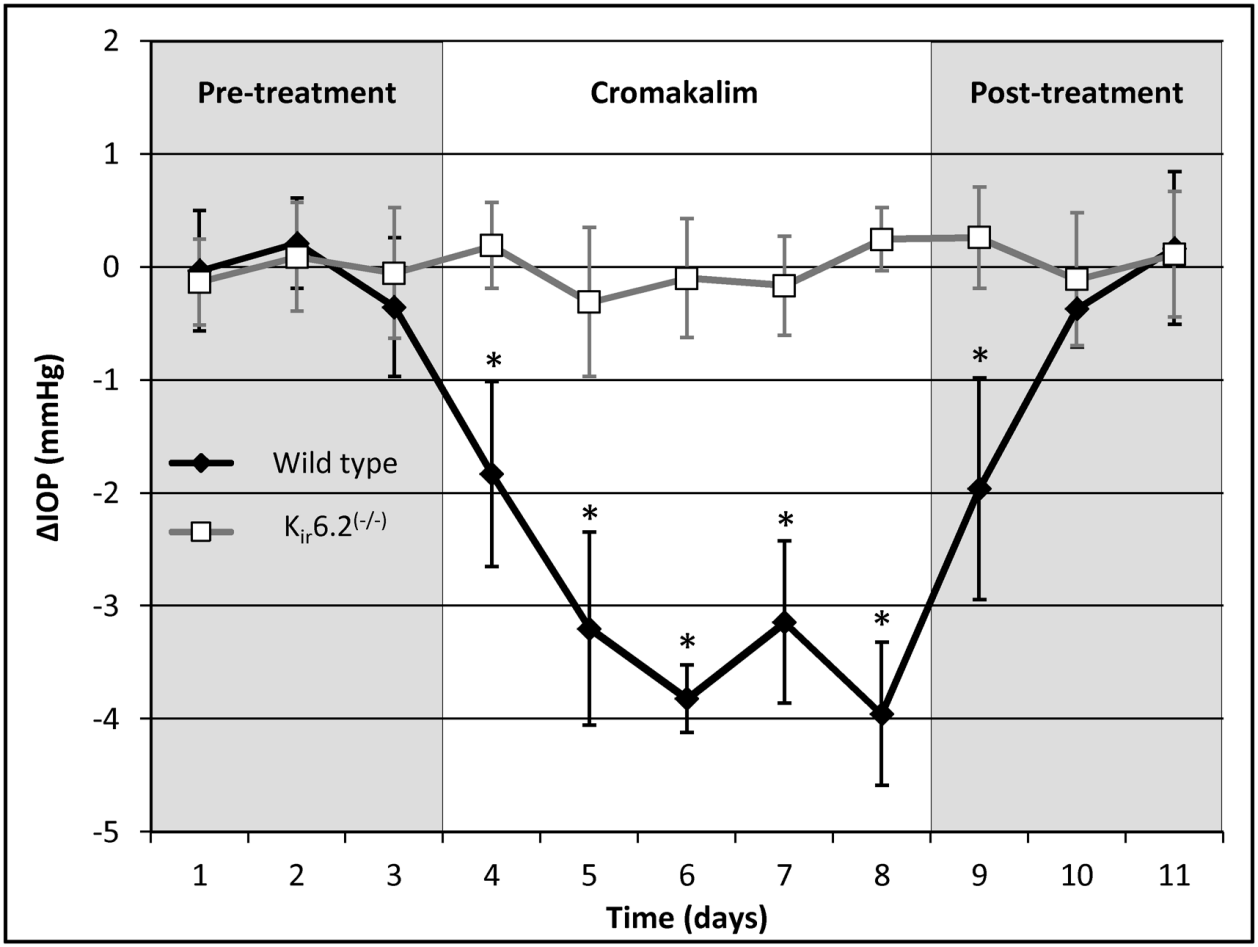
Cromakalim lowered intraocular pressure (IOP) in wild type C57BL/6 mice but not in K_ir_6.2^(-/-)^ mice. IOP was significantly reduced within 24 hours of 5 mM cromakalim treatment. Average IOP reduction was calculated to be 3.19 ± 0.41 mmHg over a treatment period of 5 days in wild type mice (n = 10). IOP was found to return to baseline levels within 48 hours of termination of treatment. In contrast, K_ir_6.2^(-/-)^ mice (n = 10) showed no change in IOP following cromakalim treatment. IOP in these animals remained at baseline levels even after topical administration of cromakalim. Values correspond to average difference (in mmHg) between treated and control eyes for individual days (ΔIOP). *p<0.01

**Fig 4:**
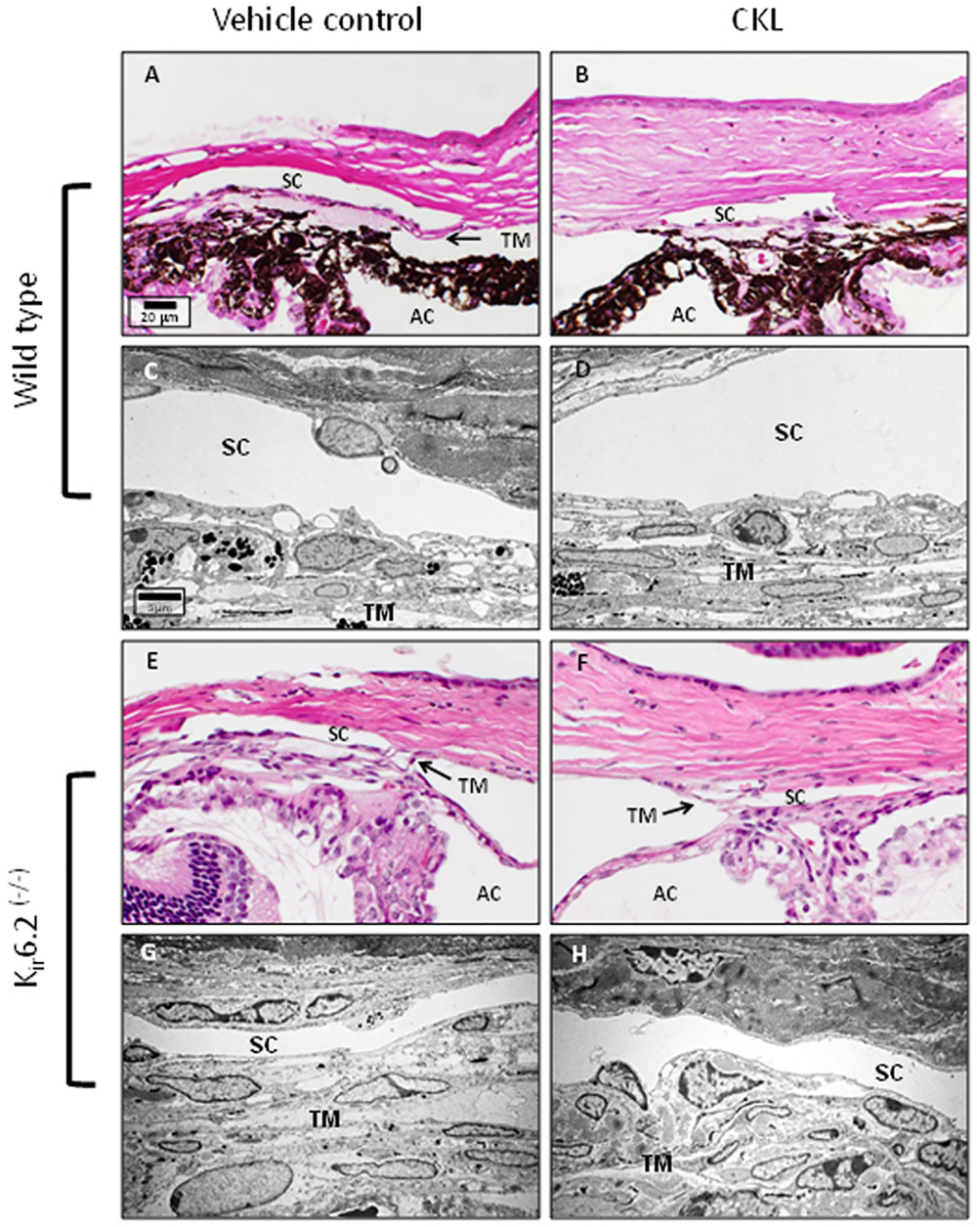
Representative images of the conventional outflow pathway of C57BL/6 (A-D) and K_ir_6.2^(-/-)^ (E-H) treated and vehicle control mice. Similar to human anterior segments, comparison between vehicle and cromakalim treated eyes showed no observable cell or tissue changes. Both vehicle and cromakalim treated groups showed normal morphology and ultrastructure with intact inner and outer walls of Schlemm’s canal, viable cells and an evenly distributed extracellular matrix. SC = Schlemm’s canal; TM = trabecular meshwork; AC = anterior chamber.

To evaluate the effect of cromakalim on IOP, wild-type C57BL/6 (n = 10) and K_ir_6.2^(-/-)^ (n = 10) mice were treated daily with 5 mM cromakalim for a period of 5 days. In the wild type mice, cromakalim treatment caused significant reduction of IOP. On average, IOP was lowered by 3.19 ± 0.41 mmHg, corresponding to 18.75 ± 2.22% reduction (n = 10, p = 0.002) over the 5 day treatment period. Within 4 hours of the first treatment, IOP was significantly lowered by 12.71 ± 5.18% (absolute reduction of 2.13 ± 0.91 mmHg). The daily reduction range of IOP was between -1.83 ± 0.82 mmHg (day 1) and -3.96 ± 0.63 mmHg (day 5) (Fig 3). IOP values for 1, 4 and 23 hour time points after treatment were all significantly lower (n = 10, p = 0.002) when averaged across the entire duration of treatment (5 days) (Table 1). Reduction in IOP by cromakalim was completely reversible with IOP returning to baseline levels within 48 hours of termination of treatment. In contrast, K_ir_6.2^(-/-)^ mice treated with cromakalim showed no significant changes in IOP between the treated and control eyes (-0.03 ± 0.14 mmHg, n = 10, p = 0.52) (Fig 3 and Table 1) even though baseline IOP in K_ir_6.2^(-/-)^ and wild type mice were similar (wild type: 16.08 ± 0.44 mmHg; K_ir_6.2^(-/-)^: 15.78 ± 0.54 mmHg, n = 10, p = 0.07). Eyes from 4 representative animals (2 wild type, 2 K_ir_6.2^(-/-)^) were histologically evaluated to determine if cromakalim treatment caused any detrimental side effects to the cells and tissues of the outflow pathway. Similar ultrastructure of the cells lining the outflow pathways of wild type and K_ir_6.2^(-/-)^ mice were found following cromakalim treatment (Fig 4A–4D). Overall, the trabecular meshwork showed viable cells with intact inner and outer walls of Schlemm’s canal. Additionally, the integrity of the extracellular matrix was found to be similar between treated and control eyes.

**Table 1:** Effect of cromakalim treatment on IOP at various times in C57BL/6 and K_ir_6.2^(-/-)^ mice.

	*Cromakalim treated* ^#^			
	Wild type C57BL/6 (n = 10)		K_ir_6.2^(-/-)^ (n = 10)	
	ΔIOP ± SD (mmHg)	% change compared to control ± SD	ΔIOP ± SD (mmHg)	% change compared to control ± SD
**1 h**	-3.01 ± 0.55*	-17.63 ± 3.19	0.43 ± 1.07	3.22 ± 8.33
**4 h**	-3.25 ± 0.39*	-19.12 ±2.07	-0.19 ± 1.58	-1.18 ± 1.58
**23 h**	-3.36 ± 0.52*	-19.65 ± 2.07	-0.14 ± 2.14	-0.14± 2.14
**Average**	-3.19 ± 0.41*	-18.75 ± 2.22	-0.18 ± 0.87	0.03 ± 0.14

#Average IOP change calculated from 5 days cromakalim.

*p = 0.002

SD = standard deviation

### Topical application of cromakalim in combination with LFA

**Fig 5:**
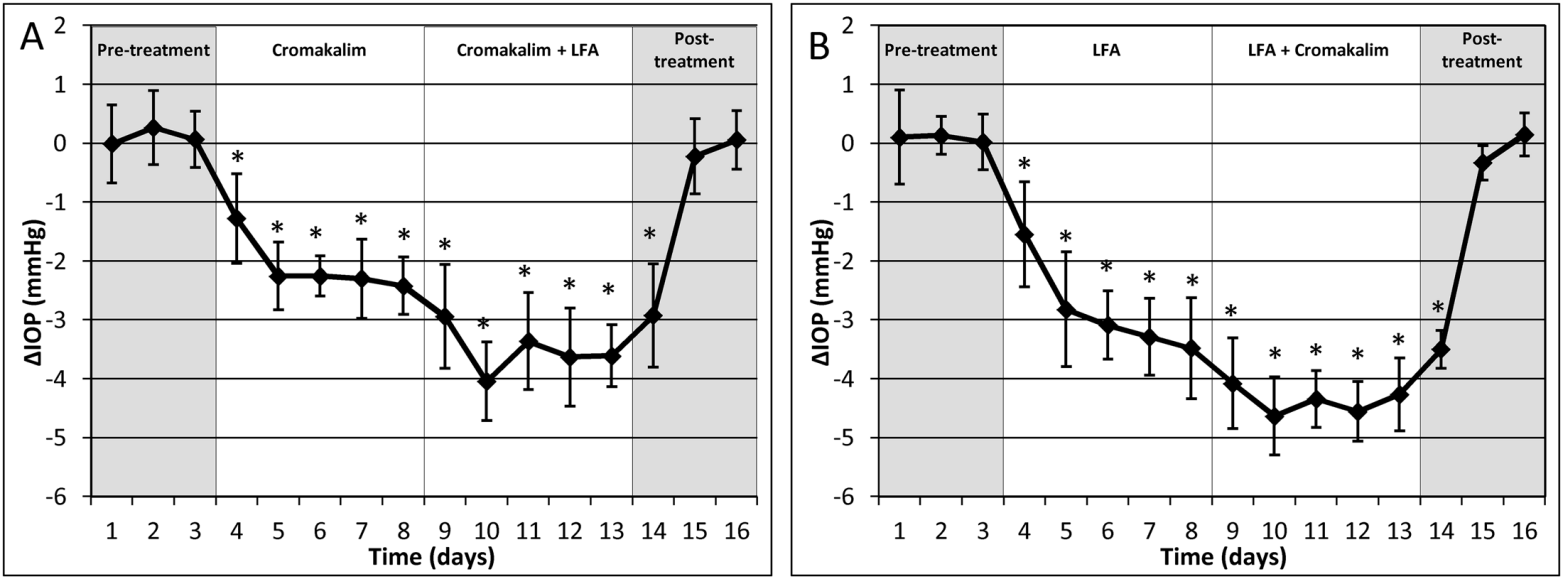
Combination treatment with cromakalim and latanoprost free acid (LFA) showed an additive effect in intraocular pressure (IOP) reduction, irrespective of which drug was used to treat the eyes first. A) Treatment with cromakalim (5 mM) + LFA (0.1 mM) showed an additional IOP reduction of 78.52 ± 47.57% (n = 10, p = 0.002) when compared to treatment with cromakalim alone. B) Combination of cromakalim + LFA showed an additional IOP reduction of 55.74 ± 19.32% (n = 10, p = 0.002) compared to treatment with LFA alone. Values correspond to average difference (in mmHg) between treated and control eyes for individual days (ΔIOP). *p<0.01.

**Fig 6:**
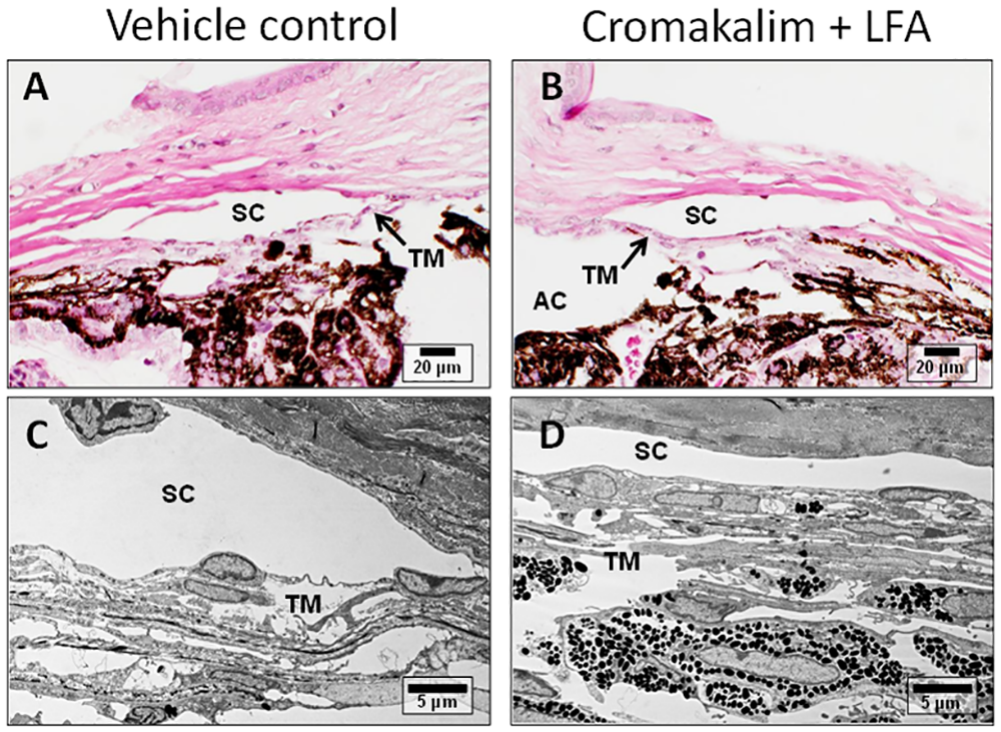
Representative transmission electron micrographs of mouse eyes treated with cromakalim + latanoprost free acid (LFA) or vehicle. Comparison of the micrographs show normal anatomy and ultrastructure in vehicle (A, C) and cromakalim + LFA treated tissue (B, D), indicating no observable changes due to treatment. SC = Schlemm’s canal; TM = trabecular meshwork; AC = anterior chamber.

To evaluate the feasibility of using cromakalim in combination with commonly used glaucoma drugs, we administered a combination therapy of cromakalim (5 mM), either alone or in conjunction with an aqueous formulation of LFA (0.1 mM). Similar to cromakalim treatment alone, the combination treatment was also applied once daily for a period of 5 days. Results showed a significant lowering of IOP following cromakalim treatment alone (-2.10 ± 0.40 mmHg, n = 10, p = 0.002). However, when these animals were treated with LFA and cromakalim, there was a significant additive effect and mean IOP was reduced by 3.61 ± 0.53 mmHg which was equivalent to an additional average reduction of 78.52 ± 47.57% when compared to eyes treated with cromakalim alone (n = 10, p = 0.002) (Fig 5A). Termination of the combination treatment resulted in IOP returning to baseline levels within 48 hours. In order to further validate the additive effect of cromakalim with LFA, 10 independent wild-type mice were treated with LFA first for 5 days followed by a combination of LFA and cromakalim for 5 additional days (Fig 5B). In these mice, LFA by itself lowered IOP by 2.84 ± 0.39 mmHg (p = 0.002). However, following combination treatment with cromakalim and LFA, average IOP was reduced by 4.38 ± 0.37 mmHg. This corresponds to an additional average IOP reduction of 55.74 ± 19.32% (p = 0.002) compared to the reduction obtained when treated with LFA alone. Histological examination by transmission electron microscopy showed no observable changes in cells and tissues of the conventional outflow pathway. The trabecular beams were found to be populated by healthy and viable cells. No breaks were observed in the inner and outer walls of Schlemm’s canal (Fig 6).

## Discussion

Target IOP in glaucoma patients is often difficult to maintain even with multiple ocular hypotensive agents. [52–54] Therefore, development of novel therapeutic agents for treating glaucoma is warranted.[55] Treatments targeting the underlying physiology of the outflow pathways will be beneficial in not only helping patients but also advancing the knowledge and understanding of the glaucoma field.[55]

In light of this, we have assessed the ocular hypotensive effects of several K_ATP_ channel openers.[46, 47] The current study shows that the broad spectrum K_ATP_ channel opener cromakalim can significantly lower IOP in human and animal model systems without any observable toxic side effects. Additionally, cromakalim was found to have an additive effect when used in conjunction with the prostaglandin analogue latanoprost. Lack of response in K_ir_6.2^(-/-)^ mice suggests that the pressure lowering effect of cromakalim is mediated through the K_ir_6.2 subunit containing K_ATP_ channels.

The molecular structure of the K_ATP_ channels comprises 4 sulphonylurea (SUR) and 4 potassium inwardly rectifying (K_ir_) subunits. These are assembled in a 4/4 stoichiometric ratio. Based on heterogeneity of the SUR and K_ir_ subunits, there can be multiple subtypes of the K_ATP_ channels. Three types of SUR subunits (SUR1, SUR2A and SUR2B) are known to pair with two types of K_ir_ subunits (K_ir_6.1 and K_ir_6.2).[56–58] However, heteromeric forms containing more than one type of SUR or K_ir_ subunit in any given channel is currently unknown in living organisms. Composition of K_ATP_ channels can differ considerably across tissues. For example, K_ATP_ channels of the pancreatic beta cells are made of SUR1/K_ir_6.2 subunits[59, 60] whereas vascular smooth muscles are mostly populated by SUR2B/K_ir_6.1 subunit containing K_ATP_ channels.[61, 62] Furthermore, variations in subunit composition are also known to impart varying degrees of sensitivity to pharmacologic openers of K_ATP_ channels. SUR2A/K_ir_6.2 subunit containing K_ATP_ channels are known to be activated by nicorandil, cromakalim and pinacidil but not by diazoxide.[63] Diazoxide is more potent in activating the SUR2B/K_ir_6.2 K_ATP_ channels along with cromakalim, nicorandil and pinacidil.[64, 65]. We have previously demonstrated the presence of SUR2A, SUR2B, K_ir_6.1 and K_ir_6.2 subunits in the conventional outflow pathways of C57BL/6 mice. Based on our earlier studies on K_ir_6.2^(-/-)^ mice, we hypothesized that the IOP lowering effect of diazoxide must be specifically mediated through the SUR2B/K_ir_6.2 subunit containing K_ATP_ channels.[47] Our current studies with cromakalim suggests a similar role for the SUR2B/K_ir_6.2^(-/-)^ channel in regulation of IOP.

In a study published by Chiang et al., cromakalim was found to increase IOP in normotensive rabbits.[50] Although this is in contradiction to our current results, several factors may be responsible for the apparent discrepancy. Cromakalim used in the Chiang study (0.5%) was almost 5 times more than the concentration we used in the current study. Also, Chiang et al. used a saline induced ocular hypotensive model where they intravenously infused 10% saline. This is an extremely high concentration of saline and it was not clear whether this was done post mortem or in live rabbits. Nevertheless, such high concentrations of saline may cause a cascade of physiologic changes any of which could be responsible for increasing IOP. Interestingly, the same study reported that cromakalim lowered IOP when administered to rabbits with experimentally induced ocular hypertension. The authors were unable to explain this convincingly. Furthermore, the authors reported using buffered saline to dissolve high concentrations of cromakalim whereas it should be noted that cromakalim is only sparingly soluble in saline and dissolves in dimethyl sulfoxide (DMSO). Therefore, a multitude of factors could have caused the reported opposite effect of cromakalim in the pressure study. The results from the current study are more consistent with what we have generally observed with K_ATP_ channel openers with reference to IOP.

Demonstration of compatibility with existing glaucoma drugs is considered an important and necessary aspect of novel glaucoma therapeutic agents.[55] Our results show that cromakalim can be used in conjunction with the commonly used glaucoma drug latanoprost since combination treatment with cromakalim and LFA produced a significantly greater reduction in IOP compared to treatment with either drug alone. The additive effect of the drugs is further validated by the fact that IOP reduction was significantly greater during combination treatments irrespective of which drug was initially used in the treatment (Fig 5). Results from the human *ex vivo* perfusion culture model indicate that cromakalim increases outflow facility and decreases eye pressure through modulation of the conventional outflow pathway. This is an important finding as no current pharmaceutical agent used to treat elevated IOP exclusively targets the conventional outflow pathway, where changes that increase outflow resistance to aqueous humor removal from the anterior chamber occurs in glaucoma.

Several models of murine glaucoma (e.g. DBA/2J or Col1a1^(r/r)^) work by physically blocking the meshwork or disrupting extracellular matrix.[7] Since cromakalim works through the conventional outflow pathway, applying cromakalim on these murine models may have been futile in the face of overwhelming physical blockage of the meshwork. Therefore, for this study, we limited our experiments to normotensive animals only. Previous reports have shown that most known pharmacological agents of glaucoma can lower IOP in normotensive mouse, rabbit and cat models.[66–70] The average IOP reduction in mice treated with cromakalim was consistent with the average IOP reduction found in these studies.

The K_ir_6.2^(-/-)^ animals used in this report is a well characterized mouse model that has been previously utilized to study cardioprotection and glucose metabolism.[37, 71–75]. We have previously used these animals to study the effect of diazoxide and nicorandil on IOP.[47] Under normal conditions, these mice are fertile and similar to the wild type in respect to behavior and body weight.[74] It may be noted that the wild type allele for K_ir_6.2 is not completely dominant over the mutant allele. As a result the K_ir_6.2^(+/-)^ mice show a phenotype that is intermediate between the wild type and the knock out mice.[76] The K_ir_6.2^(-/-)^ mice have been extensively backcrossed to the C57BL/6 mice and therefore the latter were used as wild type controls in this study.[37, 47, 77, 78] Inability of cromakalim to lower pressure in K_ir_6.2^(-/-)^ mice indicate that similar to other K_ATP_ channel openers such as diazoxide and nicorandil, cromakalim also lowers IOP by acting through the K_ir_6.2 subunit containing K_ATP_ channels. However, the exact downstream mechanisms following activation of the K_ir_6.2 subunit containing K_ATP_ channels that leads to IOP reduction is currently unknown. Cell signaling events following K_ATP_ channel activation and IOP reduction are currently being investigated.

It should be noted that all commercially available K_ATP_ channel openers are only soluble in various organic solutions. This makes them difficult to formulate into eye drops for ophthalmic use. Encouraged by their excellent ocular hypotensive properties, we are developing cromakalim prodrugs in order to make them more water soluble and therapy friendly. Cromakalim is an ideal compound since previously published structure-activity relationship studies have shown that it can be chemically modified without losing K_ATP_ channel opening activity.[48, 49]

In conclusion, cromakalim has a strong ocular hypotensive effect with no observable cell and tissue toxicity and can be used in conjunction with existing glaucoma drugs (e.g. latanoprost) to lower IOP in an additive manner. Efforts are currently underway to develop and test water soluble analogs of cromakalim. Availability of a therapy friendly version of cromakalim that can be brought to clinical trials on human subjects in the near future will be a major advancement in the field of glaucoma drug development.
