# Correction: Ocular Hypotensive Effects of the ATP-Sensitive Potassium Channel Opener Cromakalim in Human and Murine Experimental Model Systems

**DOI:** 10.1371/journal.pone.0144791

**Published:** 2015-12-15

**Authors:** Uttio Roy Chowdhury, Cindy K. Bahler, Bradley H. Holman, Peter I. Dosa, Michael P. Fautsch

The following information is missing from the Funding section: Supported in part by National Eye Institutes research grant EY 21727; Mayo Foundation, Rochester, MN; and Research to Prevent Blindness, Inc., New York, NY (MPF is a recipient of a Lew R. Wasserman Merit Award and the Department of Ophthalmology, Mayo Clinic is the recipient of an unrestricted grant).
